# Toward Cybersecurity Testing and Monitoring of IoT Ecosystems

**DOI:** 10.1007/s42979-026-05048-8

**Published:** 2026-05-20

**Authors:** Steve Taylor, Martin Gilje Jaatun, Aida Omerovic, Ravi Borgaonkar, Robert Seidl, Norbert Goetze, Jens Kuhr, Dmytro Prosvirin, Manuel Leone, Andrey Kuznetsov, Anatoliy Gritskevich, Antonis Mpantis, Oscar Garcia Perales, Bernd-Ludwig Wenning, Sayon Duttagupta

**Affiliations:** 1 University of Southampton, Southampton, UK; 2 SINTEF Digital, Trondheim, Norway; 3 SINTEF Digital, Oslo, Norway; 4 Nokia Bell Labs, Munich, Germany; 5 Antonov Airlines, Kiev, Ukraine; 6 Telecom Italia, Turin, Italy; 7 World Research Center of Vortex Energy (WRCVE), Kiev, Ukraine; 8 ATC, Athens, Greece; 9 i4RI, Valencia, Spain; 10 Munster Technological University, Cork, Ireland; 11 COSIC, KU Leuven, Leuven, Belgium

**Keywords:** Security testing, Security monitoring, IoT, Tool chains, Workflow, Software supply chain, Cyber physical systems, Systemic vulnerability, Threat and risk analysis

## Abstract

Internet of things (IoT) ecosystems introduce significant cybersecurity challenges due to device heterogeneity, firmware opacity, constrained resources, distributed deployment, and the integration of devices within wider socio-technical systems where they are used. Existing approaches to address IoT cybersecurity typically address isolated aspects of this problem, such as vulnerability enumeration, anomaly detection, or risk assessment; but without integrating them across the full lifecycle of devices and systems. This paper presents an extensible architecture that unifies cybersecurity testing, runtime monitoring, contextual risk modelling, secure update mechanisms, and auditable evidence management for IoT ecosystems that aims to address these challenges. The framework supports both device under test and system under test perspectives and integrates component-level techniques (such as SBOM generation, network fuzzing, machine learning-based anomaly detection, and access control risk evaluation) with system-level, knowledge-based, risk modelling to capture threat propagation across interconnected assets. A distributed ledger-backed auditable data infrastructure ensures integrity and traceability of indicators, results, and decisions. Automated workflow orchestration enables flexible tool chaining and lifecycle-aware execution aligned with established security development lifecycles. The approach is validated through three industrial use cases in aviation cargo monitoring, smart manufacturing, and telecommunication residential gateways. Results demonstrate the feasibility of combining static analysis, runtime indicators, and dynamic risk assessment to prioritise vulnerabilities contextually, detect anomalous behaviour, and support secure patch deployment in resource-constrained environments. The work advances lifecycle-integrated, system-aware cybersecurity assurance for IoT ecosystems and highlights the need for contextualised, interoperable tooling to address systemic vulnerability and risk propagation in complex systems where IoT, ICT and people interact.

## Introduction

Internet of Things (IoT) devices present specific challenges regarding cybersecurity: they are often low-power, highly distributed, integrated into many different systems and contexts of usage, combine hardware and software and are in many cases opaque in terms of their software construction. This paper follows Taylor et al. [1], which described challenges for the cybersecurity testing and monitoring of IoT ecosystems and proposed a high-level approach to address these challenges. Here we describe a framework, tools and methodologies that together aim to address these challenges, and this forms its key contribution: we describe how the challenges are addressed, along with key findings from experiences in developing, implementing and testing the framework architecture.

The paper is structured as follows. Next is a background and challenges section, reprising material from Taylor et al. [1], as it describes the problem statement this paper addresses. Following this, we describe the methodology and architecture of the testing framework, along with details of each tool and component within it. We then briefly describe the three use cases within the TELEMETRY project and show how the tools and the framework are used in each scenario. Following this is a section that describes the approaches to addressing the presented challenges, including key findings from experiences as a result of this work. Finally, brief conclusions summarise the results and provide pointers for further work.

## Background and Related Work

The societal and economic benefits from the rapid advance of the digital economies are at the core of the European Digital Agenda [2]. This is bolstered by the Next Generation Internet (NGI) initiative [3] with an emphasis on creating a more resilient, trustworthy and sustainable Internet for our digital future. As the Internet of Things (IoT) brings connectivity and networked intelligence to the physical things around us, highly distributed and complex infrastructures are emerging ultimately forming IoT ecosystems, which can be defined as: *systems of interconnected IoT devices with hardware*,* software*,* services and backbone network communication infrastructure to support the required system functionality*.

While these ecosystems bring many benefits and efficiencies, their inherent complexity, heterogeneity and dynamicity and distributed nature create challenges for the management of security, testing, validation, reliability and assurance at scale. The characteristics of IoT ecosystems encapsulate some key challenges for cybersecurity testing and assurance of hardware, software and service components such as: (i) IoT devices are often “black boxes” to deployers and users, meaning that their structure and inner components are not accessible for testing; (ii) IoT devices are hard to update due to the specific nature of their firmware and that the devices may be manifold and geographically distributed and (iii) vulnerabilities in one component may allow threats and risks to propagate to other components in a given system. The key challenges regarding the design of the framework from Taylor et al. [1] are summarised as follows, compared with existing literature to motivate the targets for the work reported here.

1. *There is a need to consider the full lifecycle of IoT components*—at their design time, their integration into systems, and operation of those systems. Foundational work in software security emphasises that security must be addressed across the full development lifecycle, and feedback from operational environments is essential for effective risk mitigation [4]. Ammar et al. [5] emphasise the need for security over the full lifecycle of IoT deployments crossing design, development, deployment and operation, as IoT systems are often long-term, feature distributed, resource-limited and heterogeneous devices with resulting complexity and cost of update and patching.
  At runtime, monitoring and anomaly detection are often employed for identifying unknown attacks and misuse in IoT systems. Intrusion and anomaly detection techniques based on machine learning have been extensively studied, with surveys documenting advances alongside persistent challenges related to false positives, dataset bias, and concept drift [6–8]. A recurring limitation is that anomaly detection outputs are often difficult to operationalise, particularly when models lack interpretability. Recent research therefore emphasizes explainable approaches that provide actionable insights for operators. Explainable intrusion detection systems using techniques such as SHapley Additive exPlanations (SHAP) have been proposed to improve trust and usability in IoT environments [9, 10]. These approaches align with the view that runtime detection should produce interpretable indicators that can inform risk assessment and response rather than isolated alerts.
2. *Threats and risks can propagate when components are connected together in systems*—vulnerabilities in one component can affect other components in a system. Recent studies [11] and previous work by the authors [12] highlight that IoT devices are often integrated into larger systems whose security properties cannot be inferred from individual components alone. Vulnerabilities in one device may propagate to interconnected components, leading to system-level risks that are not captured by traditional vulnerability scoring approaches that consider devices in isolation. These observations motivate lifecycle-aware and system-oriented security approaches that extend beyond isolated testing or monitoring.
  Software Bills of Materials (SBOMs) are increasingly promoted as a mechanism for improving transparency and enabling vulnerability management. Studies demonstrate the value of SBOMs for identifying component-level exposure to known vulnerabilities, particularly in critical infrastructure contexts [13]. Nevertheless, SBOM-based vulnerability enumeration alone provides limited insight into exploitability or system-level impact when deployment context is ignored. Recent work on vulnerability prioritisation shows that severity-based scoring must be complemented by exploitability and contextual information to support effective decision-making [14, 15]. Firmware composition studies further reinforce that SBOMs must be interpreted cautiously in IoT environments, motivating the integration of SBOM data with runtime observations and system-level analysis.
3. *IoT devices present limitations to current testing and management* due to geographical distribution, opacity and limited processing power. Early work on robustness testing demonstrated that mature software frequently fails when exposed to unexpected inputs [16] and subsequent attack-injection studies show that similar robustness vulnerabilities persist in modern networked systems [17], motivating the application of fuzzing and attack-injection techniques to IoT devices that process untrusted inputs under constrained conditions. However, despite their effectiveness, fuzzing and attack-injection approaches face practical challenges in IoT contexts due to limited observability and access over sometimes large distances, resource constraints, protocol heterogeneity, and the difficulty of generating representative inputs and fault models without detailed knowledge of device firmware and deployment environments.
  Firmware-centric security assessment presents additional challenges due to architectural diversity, proprietary components, and hardware dependencies. Surveys of IoT firmware vulnerability detection techniques highlight persistent difficulties related to firmware unpacking, emulation, and analysis coverage [18]. Software supply chain security has emerged as a critical concern for IoT ecosystems, which commonly incorporate numerous third-party and open-source components. Large-scale studies of firmware composition reveal supply-chain practices such as implicit patching, where vulnerabilities are mitigated without explicit version updates, complicating version-based vulnerability matching [19].
4. *Risk assessment fulfils an important requirement* because it enables assessment of what elements are important to the system’s stakeholders, how these elements may be compromised, and how the compromises may be controlled. Risk assessment and accompanying evidence documentation is also important for regulatory compliance (e.g. EU Regulation EU 2017/745 on Medical Devices [20]) and standards conformance (e.g. ISO 27001 certification [21]).
  Traditional vulnerability management approaches focus on individual components and generic severity metrics, but research in cyber-physical [22] and IoT systems [23] argues that risk frequently arises from interactions between interconnected assets rather than from individual components in isolation. Knowledge-based and system-level risk modelling approaches explicitly capture threat propagation and contextualise vulnerabilities within system architectures [12]. Such approaches enable reasoning about cascading effects and stakeholder-specific impacts that are not addressed by component-centric methods.
  In parallel, regulatory and assurance requirements increasingly demand trustworthy and auditable security evidence. Distributed ledger technologies have been proposed to support integrity and non-repudiation of security records in distributed systems [24]. IoT security monitoring research further highlights the importance of traceability and evidential quality, particularly in regulated domains such as smart manufacturing and telecommunications [25].
5. Feedback from operational monitoring of IoT devices can inform firmware updates/patches to the devices but there is a significant challenge in *rolling out these patches to multiple*, *geographically distributed low-power devices*. Empirical evaluations of real-world IoT deployments demonstrate that vulnerabilities frequently remain unpatched due to limited update mechanisms, opaque firmware composition, and insufficient continuous assessment practices [11].

### Gaps

In summary, existing research highlights persistent challenges in IoT cybersecurity related to fragmented tooling, limited lifecycle integration, firmware opacity, context-free vulnerability prioritisation, and insufficient auditability. While individual techniques address subsets of these issues, prior work does not provide an integrated approach that combines vulnerability discovery, runtime monitoring, contextual risk assessment, and trustworthy evidence across the IoT lifecycle. These gaps motivate frameworks that unify these capabilities and support continuous, system-aware cybersecurity assurance for IoT ecosystems.

**Fig. 1:**
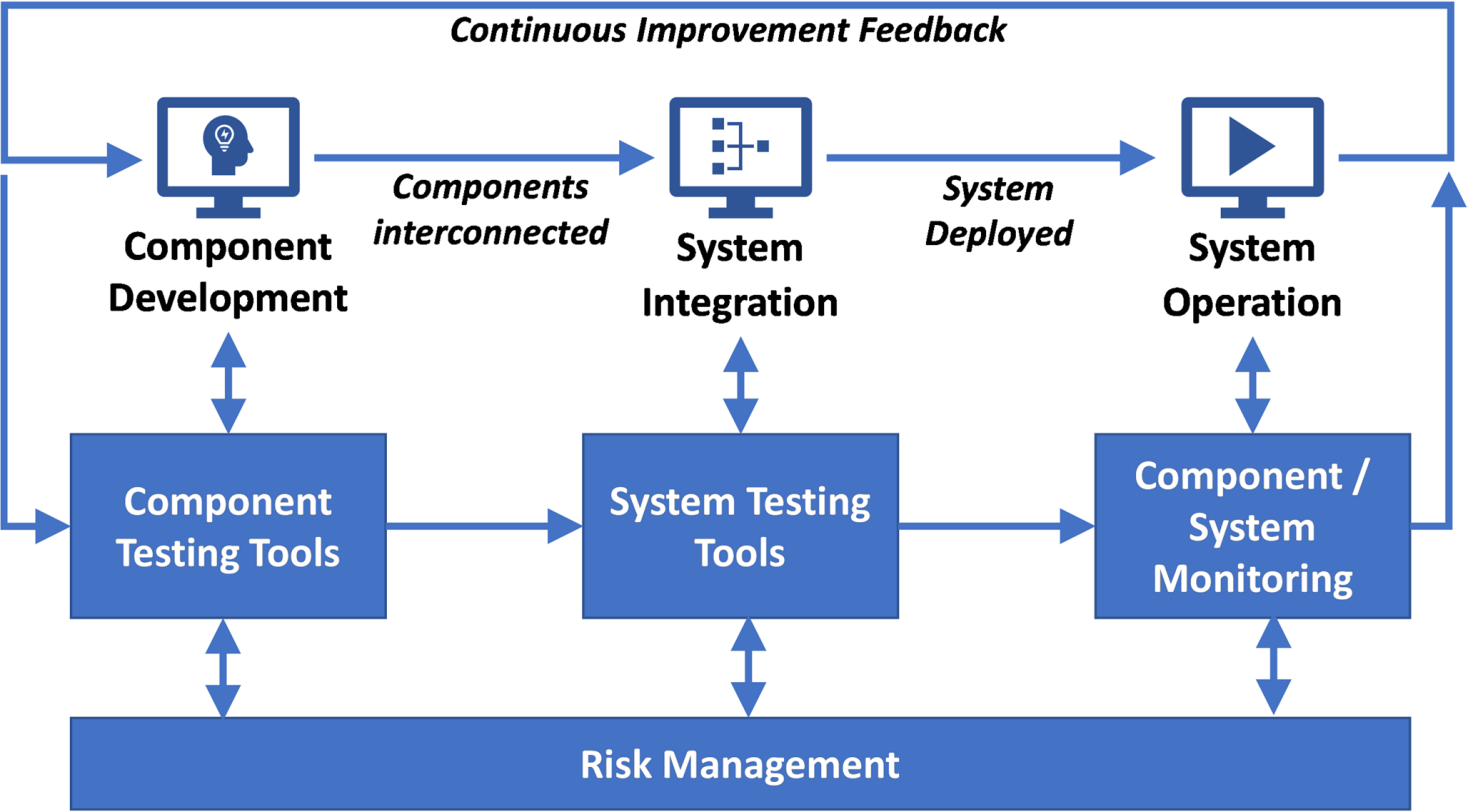
IoT ecosystem lifecycle and testing tools reproduced from Taylor et al. [1]

To address these challenges, there is a need for tools, techniques and holistic methodologies for cybersecurity testing and vulnerability detection at both component level and also in the systems the components are integrated into. This need has motivated the TELEMETRY concept, shown in Fig. 1. These elements will support continuous assessment of IoT components and ecosystems over their whole lifecycle, supporting the propagation of assurance for component developers, system integrators and operators, who act on behalf of ecosystem’s eventual users.

## Methodology and Architecture

### Support for Security Development Lifecycle

**Fig. 2:**
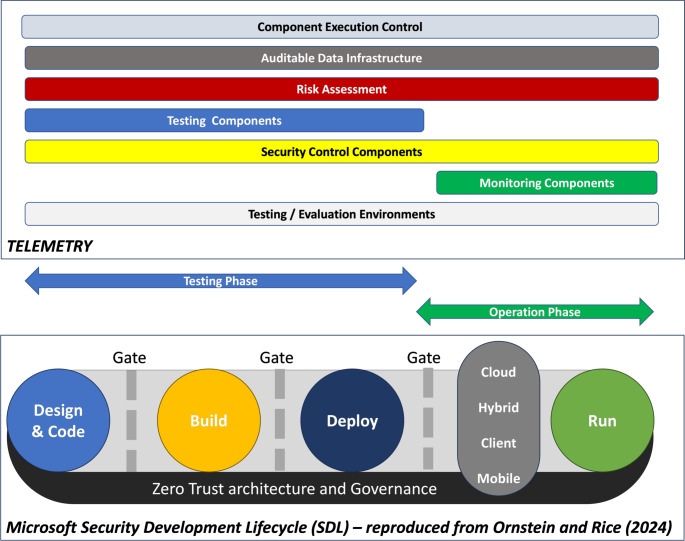
TELEMETRY Support for Microsoft SDL

The Microsoft Security Development Lifecycle (SDL) exemplifies a modern approach to security by design and in operation. The white paper on evolving the SDL by Ornstein and Rice [26] describes the lifecycle of the SDL. The concept of TELEMETRY is shown in Fig. 1 and describes a primitive of a software development lifecycle, plus the function of the TELEMETRY tools and infrastructure. This has been evolved into Fig. 2, which describes the relationship between the TELEMETRY approach and the SDL.

The early phases of the SDL are where the device is engineered (Designed and Built). Depending on the phase, the software may not yet exist (requirements, design), be incomplete (security testing), or completely (or as near as) finished (penetration testing). This can include “conformance testing” of third-party hardware before inclusion in a system. The latter phases of the SDL involve Deployment of devices into systems and operation (Running) of these systems.

### TELEMETRY Architecture

**Fig. 3:**
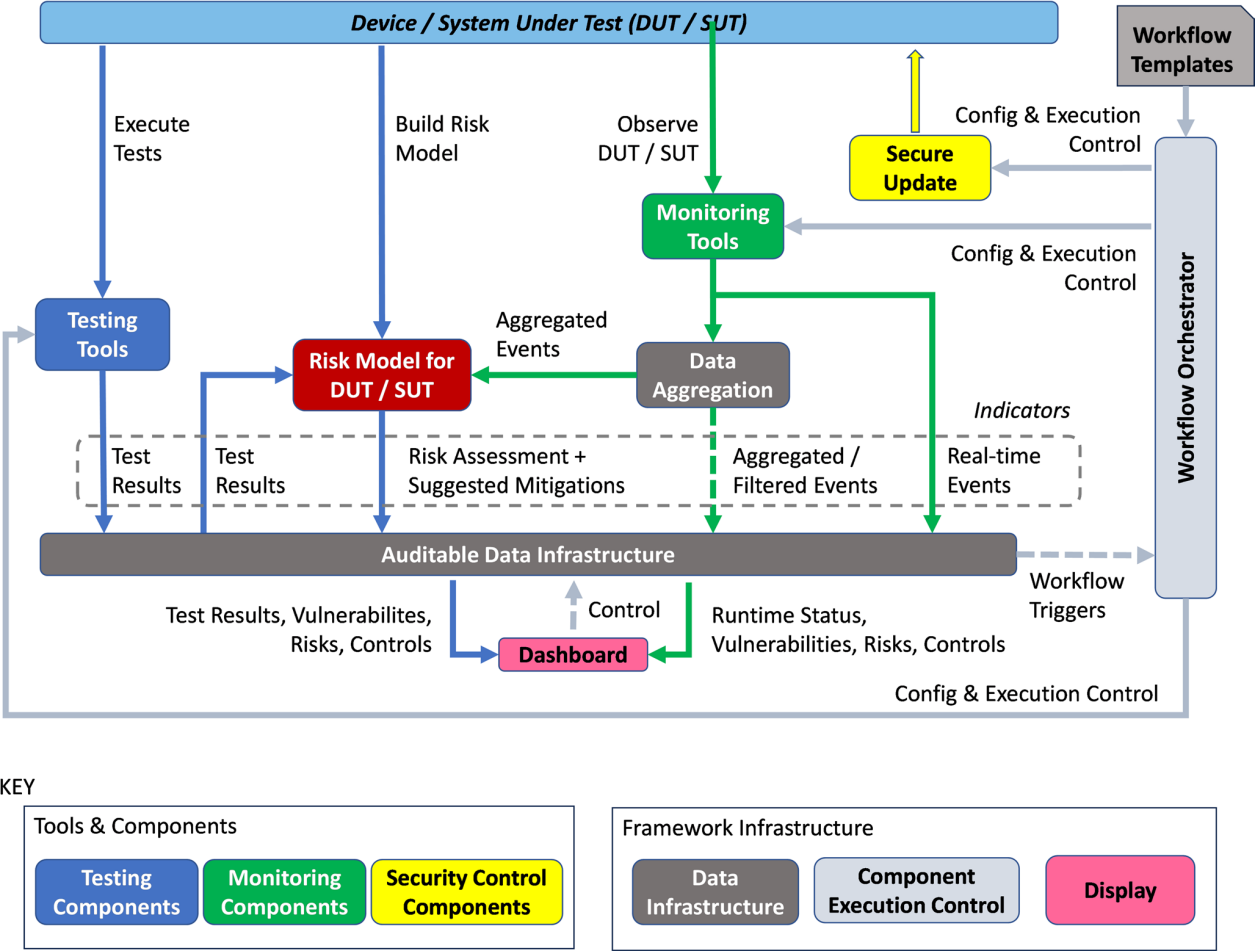
TELEMETRY conceptual architecture

TELEMETRY supports the SDL via different tools and infrastructure that operate at different lifecycle stages of the Device Under Test (DUT) and System Under Test (SUT), and the TELEMETRY Conceptual Architecture is shown in Fig. 3.

TELEMETRY provides tools in several classes as described next, but additional third-party tools may be integrated into the framework. Further details about the tools in each class are provided later.

*Testing Tools* (blue) are deliberately executed to test some characteristic of the Device / System Under Test (DUT/SUT) and with the expectation of specific outputs. These tools are typically used within the design and build phases for a device and its Deployment within a wider system, although testing may be undertaken within an operational system in the Run phase also. Examples of TELEMETRY testing tools include Network Fuzzing, SBOM generation and Access Control testing. 

*Monitoring*,* Analysis and Detection Tools* (green) observe the DUT/SUT as it is operating and raise events if specified conditions or anomalous conditions occur that enable us to detect bugs and flaws that we can feed back to the development phase for rectification—“feedback from the field” [4]. These tools are predominantly used during the Run phase of a system with devices and may generate events that can be communicated to other TELEMETRY tools, such as Risk Assessment or inform additional testing. Such tools may include post-deployment penetration testing tools, intrusion detection tools, and various monitoring tools.

*Security Control Components* (yellow) may be applied to the DUT/SUT to manage risks identified. These components may be used at any stage of the SDL from Design to Run, depending on the type of control. There are many examples of controls, from best practices (e.g. training and procedures) to technical measures (such as encryption or software patching), and TELEMETRY’s focus in this area is focused on a technical mechanism for distribution of updates. This is primarily focused on the “Run” phase of the SDL because it involves live roll-out of software updates to distributed IoT devices.

*Risk Assessment* (red) evaluates the impact and likelihood of compromises detected by the testing or monitoring and detection tools on the DUT or the SUT, along with recommendations of controls, if the resulting risk level is unacceptably high. This can occur at any phase of the SDL and may consider device as a system in its own right, where, for example, vulnerabilities in third party libraries or hardware affect the output of bespoke code. This is more likely in the earlier stages of the SDL, i.e. Design or Build. Risk assessment may also consider systems of devices at deployment time, i.e. when the system of devices is constructed, in which case, risk assessment is executed as a testing tool to assess the systemic risk before deployment. Alternatively, risk assessment may operate at Runtime where it may be informed by the output of monitoring or detection tools, to reactively compute any change in the risk of the operating device or system based on detected events.

*Auditable Data Infrastructure* (dark grey) provides means to gather events, to aggregate those events, for tools and other components to securely and auditably exchange information and to interact with testing users. This is a key infrastructural element that operates at all phases of the SDL, as it supports both testing and monitoring/detection.

*Display* (pink) —a dashboard enables a user to observe events, test results, risk assessments, etc. The dashboard is connected to the Auditable Data Infrastructure.

*Component Execution Control* (light blue-grey) is infrastructure that enables tools and other components to be configured and executed in different operational sequences depending on the needs of the situation at hand. This will be applicable at all phases of SDL.

*Indicators* represent information of relevance for assessment of cyber security risk, such as observable vulnerabilities, incidents and threats. Indicators are discussed more fully by the authors in Taylor, et al. [27] but briefly, indicators have the following properties. Indicators are all types of signal that may trigger an action or be used as evidence in a decision. Indicators can come from each tool and may be used as input for another. Indicators may be aggregated to provide empirical evidence, which is a composition of several pre-defined indicators, for example, one event may not generate an important alarm, but the conjunction and correlation of different events cause an important alarm to be raised. Indicators are likely subject to change over time, due to a change in the SUT/DUT. The changed values need to be captured in timely way, in order to provide up to date information, and the trend over time may in itself be an indicator. Moreover, indicator values may need to be measured on demand in order to confirm an unchanged or assumed value (or state) and thus reduce uncertainty of a risk model.

### Device Under Test vs. System Under Test

A key element of TELEMETRY’s concept is the relationship between Devices and Systems, as they are the subjects of testing and monitoring of TELEMETRY. For the scope of TELEMETRY, when we consider a Device, it may be a software component, or an IoT device with hardware and firmware. Also, for the scope of TELEMETRY, the term “System” may be defined as *“a set of connected things or devices that operate together”*^1^. This implies that Systems are composed of Devices, which is certainly true, in that a Device may be interconnected and interact with other components in a wider System, for example an IP camera working in a manufacturing environment interacting with a manufacturing robot in a smart factory System. However, given that IoT and software components are themselves composed of sub-components, e.g. hardware, firmware and software, which is highly likely to be built from third party libraries, it is reasonable to consider Devices as Systems in their own right, and the terms “Device” and “System” are applicable from the perspective of the context of concern and visibility. We therefore use the terms “Device Under Test” (DUT) and “System Under Test” (SUT) to describe the subjects of concern. In summary:

- A system is an interconnected set of components.
- A device can be a component within a larger system.
- A device can be a system in its own right.

In some cases, a Device is a black box (e.g. when an IP camera is a device bought from a supplier and deployed in a smart factory system—as in TELEMETRY use case 2—see later). We are often unable to understand, monitor or control the inner workings of the device and in many cases, we have no alternative but to trust the manufacturer. Devices such as IP cameras are often cheap commodity goods with no guarantees of freedom from vulnerabilities or of any software updates should vulnerabilities be discovered. This has given rise to one of the challenges from Taylor et al. [1]—that the IoT devices are deployed within systems we are concerned about, but we have often little control over their vulnerabilities, and they may compromise other devices in the system. The notion of Software Bills of Materials (SBOMs) is certainly helpful here, as it helps a system deployer to understand the software composition of a device within their system, but the critical challenge is that SBOMs are often not provided and are difficult to create by the user for some devices. We may have control over other aspects of the system, for example we can monitor the network traffic from the IP camera and look for anomalous behaviour such as data transmission to unexpected destinations and block them if detected.

In other cases, if we are building the IP camera, we are concerned with its composition of its sub-components, e.g. third-party libraries, bespoke code and hardware and their interactions so as to perform the function of the camera. Here, we are considering the IP camera as a System in its own right. If we have this degree of visibility, especially if we are the creator of the device, we will have made decisions about, and therefore know better about, the supply chain of software and hardware that the device is composed of.

## Framework Tools and Components and Methods

The tools and components of the TELEMETRY approach are described in this section.

### Testing Tools

Testing tools (blue in Fig. 3) can be executed by the user on demand and produce results that can be interpreted by the user or fed into other tools for further analysis.

### Network Fuzzer

The network fuzzer (as initially proposed by Miller et al. [16]) facilitates security testing by sending specifically crafted requests to the interface under test and observing whether it responds or behaves in an unexpected way.

**Tool Name**: Network fuzzer. **Purpose**: The network fuzzer facilitates security testing of network interfaces, by assisting with the detection of unknown vulnerabilities. **Features and Benefits**: The main benefit of the tool is that it allows for an automatic detection of unexpected behaviour. By using this as a starting point, security analysts can greatly improve the efficiency of searching for new vulnerabilities. The tool builds on the open source boofuzz^2^, which provides a well-documented framework for network fuzzing. Key extensions involve enabling the tool to generate test cases based on network captures from the interfaces of interest, reducing the need for manual configuration and set up, and implementing a standardized way of delivering/presenting test results. **Input**: A packet capture (.pcap) file^3^ from the device or interface to be tested. This file shows the packets and protocols involved with communication to the interfaces which we would like to test. **Output**: The output of the tool should be a set of tuples, containing a description of the observed behaviour and an associated packet capture file with the packets required for reproducing the reported behaviour. **Results**: We have employed the network fuzzer in a Residential Gateway (e.g. domestic router), both on the generic OpenWRT firmware and enhanced firmware (based on OpenWRT) supplied by the RG vendor. It is acknowledged that the tool requires significant manual configuration effort on part of the tester but enables significant insight to be gained resulting from differences between OpenWRT and the commercial Residential Gateway, where an additional service in the latter was crashed by fuzzing, thus exposing a previously unreported vulnerability.

### SBOM Generator

A Software Bill of Materials (SBOM) is a structured overview of all external libraries or software components used to build a software program/system (see Jaatun et al. [13]). There are currently three major SBOM formats: SPDX^4^ from the Linux Foundation; CycloneDX^5^ from OWASP; and SWID as defined in ISO/IEC 19770-2^6^. The general idea is that software developers should create a distinct SBOM for every version of their software that they publish, enabling customers and users to quickly determine whether, e.g., a given vulnerability applies to the version they are using.

Even though provision of SBOMs has been mandated by the US Government^7^ and mentioned in EU legislation^8^, they are still far from ubiquitous and many software packages lack SBOMs. We have thus explored to what extent we can create an “aftermarket” SBOM based only on analysis of a binary executable or firmware. We also map the discovered versions to the National Vulnerability Database^9^ to flag any relevant vulnerabilities. This mapping can be performed periodically to catch any newly discovered vulnerabilities.

The SBOM generator gives an overview of the software components and libraries included in a software product. This will in turn allow the tool to list known vulnerabilities present in the software product and the vulnerabilities’ severity. The tool makes use of a virtualized infrastructure, enabling it to operate without the need for physical hardware. This “digital twin” is constructed using the open-source quick emulator (QEMU) package^10^, which offers a versatile framework for emulating diverse hardware platforms. This framework is enhanced by facilitating the tool’s ability to effortlessly replicate IoT device configuration files and Integrating vulnerability scanners to detect and analyse security vulnerabilities effectively. Firmware imported to QEMU is analysed using the “binwalk” tool^11^, and the extracted files are manually studied to determine the root file system. Printable strings are extracted to help identify libraries used.

**Tool Name**: SBOM generator. **Purpose**: Provides an overview of the software components and libraries included in a software product. **Features and Benefits**: Having an overview of components in a software product is a prerequisite for determining which vulnerabilities affect the SUT, which in turn is essential when performing risk assessments or vulnerability management. While an SBOM is normally generated during the build process of a software product, in many cases this does not occur, and this tool seeks to perform SBOM generation after the software has been released, only using the software package or binary file. **Input**: The input to the tool is a binary file or a software package, in the same format as it is delivered by the software development company. **Output**: The output of the tool is an SBOM file with associated vulnerabilities represented as CVEs. **Results**: The tool performs reverse engineering of the binary file by deploying it into an emulation environment and decomposing it using e.g. package manager software with subsequent mapping of libraries and versions to CVEs via queries to the National Vulnerability Database. Where a vendor-supplied SBOM exists, the tool has the additional benefit of checking the validity of this via comparison, and the CVE lookup can be employed with both existing and generated SBOMs. The process has certain limitations, in that generation and coverage of the SBOM is dependent on whether the source is obfuscated or not.

### IT Infrastructure Access Control Risk Evaluation Using Fuzzy Logic

Complex IT infrastructures may require a single comprehensive access control solution. This is especially relevant for networks that were scaled unevenly, without a pre-designed architecture, with a change in the main responsible in the process, as well as several software and administrative solutions that do not make up an optimal and coordinated system. A methodology is proposed, according to which Subjects (users) and Objects (services) are evaluated according to significant factors and with the help of a mathematical model based on fuzzy logic, the risk of providing access is assessed. Bakurova et al. [28] and Lytvyn et al. [29] provide full details of the approach, which is intended to help make more informed situational or system decisions for access management. Subjects are evaluated according to such factors as authentication level, access level, abnormality of behaviour, etc. Objects are represented by such factors as the level of vulnerability, the frequency of access, the level of data sensitivity, etc. Interaction factors such as attack vector or network type are also suggested. These are all combined, to determine risk levels considering probability of an attack and the level of seriousness of the attack.

**Tool Name**: Access Control Risk Evaluation Using Fuzzy Logic. **Purpose**: To evaluate access control risk of failure. **Features and Benefits**: The mathematical model’s basis in fuzzy logic allows consideration of ambiguity in the description of the state of the system and assessments of its vulnerabilities. Combining modern network monitoring tools, vulnerability libraries and comprehensive IT infrastructure data analysis using fuzzy logic outperforms existing approaches that lack any of these components [29]. **Input**: Influencing factors, indicators or events observed from logs or other TELEMETRY tools such as frequency of access to objects via monitoring logs from servers and network equipment. **Output**: Risk level (high/medium/low) based on combination of factors, such as the criticality and vulnerability of objects. **Results**: The risk assessment helps to improve access policies and the access control system itself, including at the human-machine level, via estimation of how exposed an object is. Specific results for the access control system analysis within a smart factory context (Use Case 2) are provided in Zaritskyi et al. [30].

## Runtime Monitoring, Analysis and Detection Tools

Monitoring tools (green in Fig. 3) aim to be installed at the location of the DUT or SUT and observe behaviours, raising events as indicators which can be fed to the dashboard for display to the user or fed into other tools for further analysis.

TELEMETRY uses a Cyber Range in the form of a commercial platform developed by AIRBUS^12^ to evaluate and test the monitoring tools via interactive cybersecurity hybrid environments that can include both virtual and physical components. The Cyber Range can generate a variety of simulated traffic and attack patterns that can be applied to a physical or a simulated SUT. Test scenarios and attack patterns will be defined for the TELEMETRY use cases. The Cyber Range will be used to run tests on simulated SUTs that have TELEMETRY network anomaly detection tools deployed in order to assess whether the TELEMETRY tools are able to correctly detect and respond to attack patterns, thereby providing tool validation beyond the scope of the use case testbeds.

An overview and initial results regarding the TELEMETRY anomaly detection tools is provided in Arguello-Ron, et al. [31] but specifics regarding each tool are discussed below.

### Nokia Anomaly Detection Pipeline

The ‘Nokia Anomaly Detection Pipeline’ (NAD) aims to ingest sensor readings from IoT devices to predict anomalous behaviour of these devices in near real-time. It is based on training a machine learning (ML) model during normal operation of the DUT that describes expected sensor readings from the DUT: i.e. a fingerprint of the device. When this ML model is then applied during operation, any deviation from the expected sensor readings can be detected and reported.

**Tool Name**: Nokia Anomaly Detection Pipeline. **Purpose**: Detects anomalous behaviour in DUT. **Features and Benefits**: This tool takes a fingerprint of recorded sensor readings from the DUT operated during normal operation and can detect abnormal situations during live operation. The Pipeline uses past sensor measurements of a DUT operating in normal situations to make predictions of expected behaviour during live operation and compares the prediction (target) with the measurement (actual) and reports significant deviations in near-real-time to downstream components. **Input**: Numeric values of measurements from the DUT reported in regular intervals. **Output**: Alarms containing information on when an anomaly is detected mapped to an alarm condition scale indicating how long the anomaly persists. **Results**: The NAD pipeline has been verified in the Smart Manufacturing use case (UC2, covered later), when monitoring the sensor readings of a robot in operation. We can detect anomalies in the reported speed and angles of robot joints. We identified irregular gaps in the robot data stream which would be reported as false positive anomalies. To address this, we sample the robot data over a timeslot 2–3 s, which produced behavioural data with no gaps using time-series processing algorithms.

### Misuse Detection ML Toolkit

The *Misuse Detection ML Toolkit* is a set of runtime libraries that includes several AI/ML algorithms with the capability of training ML models to detect the misuse by humans of software components and systems, developing ML for Intrusion Detection [8, 32] towards misuse detection based on baseline normal behavioural patterns. Convolutional Neural Networks (CNN) are used to identify these patterns in historic usage scenarios such as normal activities of users and/or similar patterns on log files or data storages. The ML will learn from user-interaction and the detection of divergences in user behaviour from the norm, using similar principles to social engineering for capturing user aspects such as user functional footprint, temporal behaviour and statistical data distribution.

**Tool Name**: Misuse Detection ML Toolkit. **Purpose**: To train ML algorithms and execute the trained ML models so that the misuse of software components and systems can be detected. **Features and Benefits**: The Toolkit allows users with little or no analytical knowledge to train their own models by following a wizard approach that will guide the user across screens and options depending on the algorithm selected. **Input**: Training data on normal usage of the DUT/SUT, for the creation of a model to recognise misuse. At runtime the model receives sensor-related data from the DUT/SUT. **Output**: Runtime alerts referring to anomalies detected corresponding to misuse. **Results**: Training data from the Smart Manufacturing use case (UC2) sensors has been gathered to characterise normal behaviour of the robot, and anomalies that were fabricated by testers have been used to validate the training of the ML model. The next development to be included here will be the ability to read SHAP-based charts that explain how the model works so that the final user is able to understand the trained model.

### r-Monitoring: Monitoring and Analysis of System Processes, Metrics and Network Traffic

The tool aims to enhance system security by providing comprehensive monitoring and analysis of system processes, metrics, and network traffic following patterns suggested by Casola et al. [33] and Shao et al. [34]. It includes dynamic file monitoring capabilities, which track changes to critical system files and directories in real time. Any unauthorized or suspicious modifications are flagged and alerted to the system administrator as these could be indicative of malicious activities. Additionally, the tool continuously scans and evaluates running processes against known malware signatures and anomalous behaviour patterns to identify potential threats.

**Tool Name**: r-Monitoring Tool. **Purpose**: Comprehensive monitoring and analysis of system processes, monitoring metrics, file monitoring. Signature checking and network traffic. **Features and Benefits**: *Resource Monitoring* (CPU and memory consumption); *System Monitoring* (detailed process consumption); *File Monitoring* (monitor sensitive files for changes); *Hash Monitoring* (check hashes for altered processes); *Network capturing and Monitoring* (signature monitor); *Anomaly Detection on Network Data*; Record *Historical Data*; output to Dashboard. The agent designed for collecting metrics, system parameters, and network traffic boasts a lightweight architecture that is compatible with various CPU architectures. Its efficient design makes it especially well-suited for devices with constrained resources (e.g. IoT), ensuring broad applicability without compromising performance. While several tools on the market provide some of the features listed above (e.g. Casola et al. [33]), it is rare to find a single tool that encompasses these capabilities comprehensively and even more for devices with constrained resources. **Input**: configuration in terms of system metrics, file status, network traffic to be monitored by the agent. **Output**: system metrics, assessments of unauthorized or malicious modifications and activities, and alerts for abnormal behaviour of system critical parameters. **Results**: The monitoring agent has been successfully developed and tested on IoT devices, specifically the ZTE router provided by the Telecoms Use Case (UC3). This agent effectively captures key metrics and network traffic data, which it then forwards to the monitoring application for further analysis.

### r-Anomaly Detection

This tool is designed to monitor network traffic and identify unusual patterns that deviate from established norms, using intrusion detection approaches exemplified by Sommer and Paxson [6] and Fuentes-García et al. [35]. It utilizes an algorithm to continuously compare incoming traffic data against a baseline dataset representing healthy or typical network activity, the tool efficiently flags anomalies, which could indicate potential security threats or system failures.

**Tool Name**: r-Anomaly Detection. **Purpose**: This tool is designed to monitor the network and to enhance network security and reliability by promptly flagging potential issues. **Features and Benefits**: Identify patterns that deviate from the norm (as established by a provided dataset of typical activity) and pinpointing the specific features that contribute to each detected anomaly (e.g. Lundberg and Lee [36]). It employs machine learning algorithms (see e.g. Wang et al. [7]) to identify deviations and the underlying causes of these irregularities, that may suggest security threats or system malfunctions. **Input**: Network traffic, which consists of data packets, each containing parameters from various layers. **Output**: JSON object that contains three values: one indicating whether an anomaly was detected, one describing the severity of the deviation from the defined baseline and a third defining the list of parameters that are considered abnormal. **Results**: We have analysed data provided by Smart Manufacturing (UC2) and Telecommunications (UC3) over network data characterized by a variety of protocols, each with distinct features. Exploring and investigating methods to handle this diversity and anomalies occurring in each case presents a significant challenge. Tackling this challenge has taught us the importance of flexibility in model design to adapt to varied data types. Our pursuit to develop a robust model has emphasized the need to investigate advanced analytics and machine learning techniques. These methods are crucial for effectively handling and interpreting diverse network traffic, and for assessing its divergence from baselines, with the aim of improving reliability and consistency in our outcomes.

## Risk Modelling of System/Device Under Test

Risk Modelling (red in Fig. 3) allows simulation of risks, which relate indicators to threats that cause harms and the priorities of harms from the perspective of different stakeholders.

### Spyderisk System Modeller (SSM): Risk Assessment

**Fig. 4:**
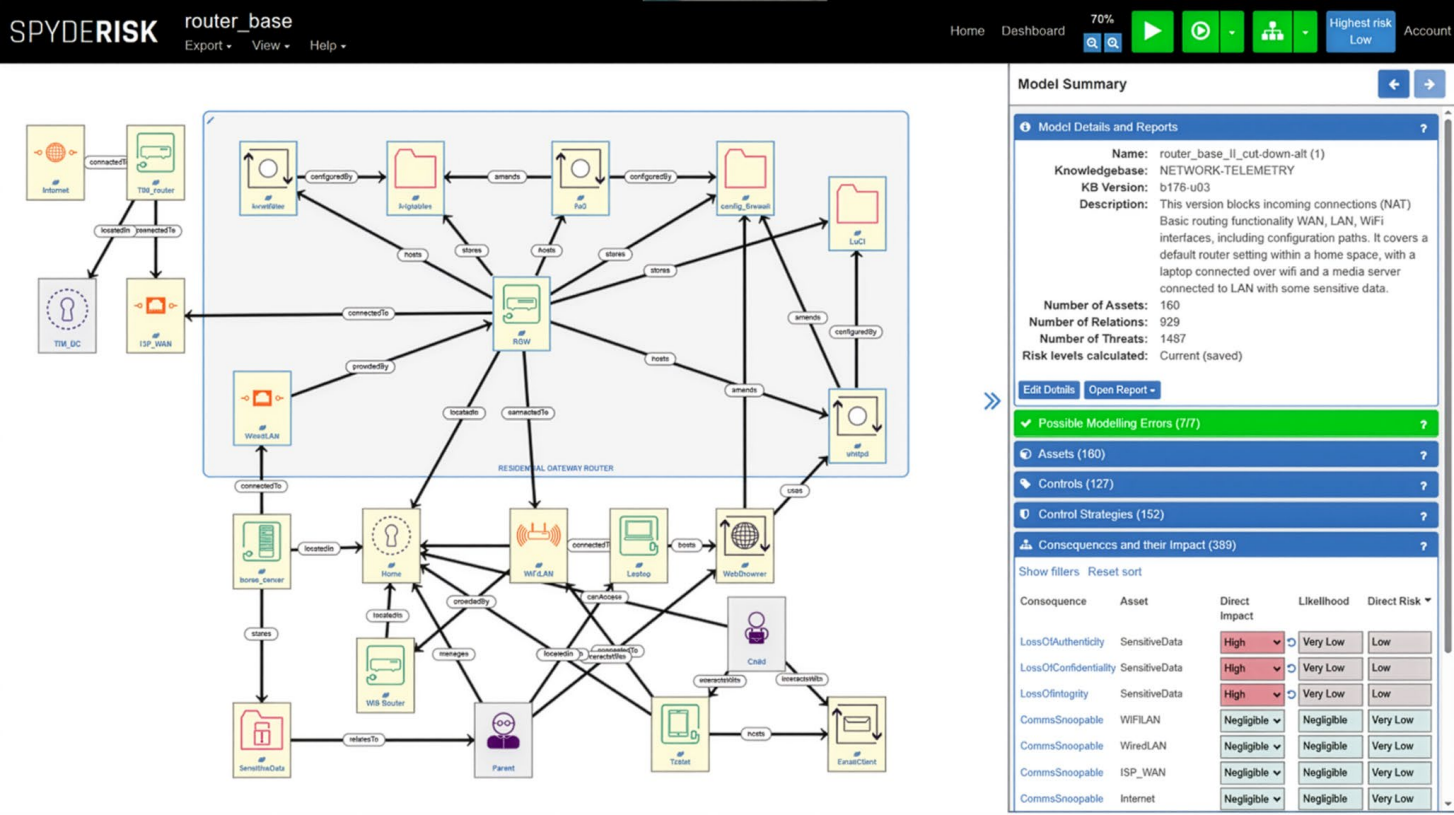
Risk modelling user interface

The Spyderisk System Modeller, abbreviated to SSM [12] is a comprehensive automated risk management toolkit designed to enhance a system’s security via the assessment of risks and recommendations of controls to lower the likelihood of risks with an unacceptably high level. SSM enables users to construct detailed system models (models of the SUT/DUT), identify cybersecurity and compliance risks, and determine the most effective mitigations. Its user interface is shown below in Fig. 4.

A panel of asset icons is provided (not shown) from which a system model can be constructed in the main window, and at the lower right, individual risks (named Consequences) are shown. Each risk can be explored to determine threats that cause it, and controls are recommended by the tool. The controls can be selected and the risk levels re-calculated to see the effect of the controls on the risks. The SSM also supports dynamic cybersecurity risk assessments that adapt to new threats as they are detected by tools like vulnerability scanners (e.g., Wazuh^13^), plus other tools developed in TELEMETRY such as anomaly detection, fuzzing or the event pipeline. This adaptive feature enables the assessment of emerging risks at runtime along with by dynamically recommending and implementing necessary security controls, so they be addressed in a timely manner, enabling the maintenance of a robust defence.

**Tool Name**: Spyderisk System Modeller (SSM). **Purpose**: A comprehensive automated risk management toolkit designed to enhance a system’s security via the assessment of risks and recommendations of controls to lower the likelihood of risks with an unacceptably high level. **Features and Benefits**: It is a knowledge-based risk assessment tool enabling system-level risk assessment at both design time and runtime. SSM system model concepts are compliant with the ISO standards 27,000/27,005. SSM enables users to construct detailed system models (models of the system under test), identify cybersecurity and compliance risks, and determine the most effective mitigations. Knowledge of vulnerabilities, threats, risks and controls to manage risk are encoded in a knowledge base designed to support automation using a cause-and-effect approach to risk modelling. The user constructs a model of their system under test, and the tool uses its knowledge base to identify relevant risks and threats and calculate risk levels, and to recommend controls to lower the likelihood of risks. Spyderisk is a simulator of the causes and effects of cyber threats in interconnected systems. Dynamic context and threat propagation via interdependencies of assets and consequences offer a continuous monitoring and updating of risk assessments as new threats emerge or as changes occur in the system. **Input**: model of SUT/DUT, state reports from SBOM generator about system vulnerabilities, typically including CVE (Common Vulnerabilities and Exposures) and CVSS (Common Vulnerability Scoring System) metrics. **Output**: a JSON object that presents the current status of the risk model and includes a list of potential recommendations. Each recommendation specifies the controls that need to be implemented and details the expected risk reduction resulting from the implementation of these controls. **Results**: SSM is deployed in Smart Manufacturing (UC2) and a process of mapping the indicators from tools such as the anomaly detection tools to vulnerabilities in components in the risk model is underway. The SSM is also deployed in Telecommunications (UC3), where a risk model of the DUT (a domestic modem-router, known as the Residential Gateway) has been created. It is necessary to consider modelling devices as systems, and a model of the router DUT has been created as a system in its own right, comprising constituent components of the router such as NAT, routing tables, switching hardware, DNS/DHCP, firewall, Wi-Fi module, ethernet connection, http server for configuration, etc. This work has been informed by the SBOM generation tool that has analysed the router DUT and provided an SBOM. The SBOM provides information of libraries and components with versions, which can then be linked to specific vulnerabilities in the CVE database. There is a need for mapping of the functional blocks in the risk model to the libraries/components from the SBOM and to understand the parameters in the risk model that are undermined should a CVE be detected.

## Security Control Components

Security Control Components (yellow in Fig. 3) aim to address risks assessed in the SUT/DUT.

### Secure Software Updates

This tool performs efficient and secure software updates of the SUT/DUT, utilizing lightweight cryptographic algorithms and novel protocol solutions to develop a new framework for secure patches. Given the diverse range of communication protocols and the challenges associated with lightweight devices, our approach focuses on minimising computational overhead while ensuring the highest level of security. This tool will be crucial in maintaining the integrity and functionality of systems in decentralised environments, where traditional methods fall short due to the limitations of low-power and low-bandwidth networks.

**Tool Name**: Secure Software Updates. **Purpose**: Provides a robust and efficient mechanism for performing secure firmware and software updates on distributed and resource-constrained (e.g. IoT) devices. **Features and Benefits**: The tool ensures that software patches and updates can be distributed and applied quickly and securely, even in the most resource-constrained environments. Unlike existing solutions, which may focus on either security [37] or lightweight operation [38], this tool achieves both. It accomplishes this by employing innovative cryptographic protocols specifically designed for low-power and low-bandwidth scenarios. Additionally, it addresses the challenge of dynamic access control, which is often overlooked in traditional update mechanisms, by allowing for adaptable permissions that can evolve as the network grows or changes. **Input**: The tool receives inputs from various components within the TELEMETRY architecture, including device status reports, update packages, and security requirements. These inputs are processed to ensure that only validated and authenticated updates are distributed across the network. **Output**: A secure, encrypted update package that can be deployed across the network with minimal disruption. This tool will be deployed in the form of a cryptographic library, which can be easily called during run-time. **Results**: Initial prototypes of the cryptographic library that will underpin the Secure Software Updates tool have been developed, focusing on optimising the balance between security and computational efficiency. Tests have been performed using simulated IoT environments to refine the tool’s performance under various network conditions and device constraints. Developing a secure update mechanism for resource-constrained devices has highlighted several challenges [11]. One key lesson is the importance of adaptability: our approach must be flexible enough to handle diverse network conditions and device capabilities without compromising security. Additionally, managing the trade-off between security and efficiency remains a constant challenge, particularly in ensuring that the cryptographic algorithms used are both lightweight and robust.

## Framework Infrastructure

### Auditable Data Infrastructure

As shown in the TELEMETRY architecture above, an Auditable Data Infrastructure is an important element for data sharing among the tools, and as a place where a record of events, test results and actions is maintained. With the variety of tools contributing to the overall TELEMETRY solution, the data infrastructure serves as a common repository where records are being kept of what was reported by the tools. In order to ensure that the records are reliable, trustworthy and meet requirements for auditability, they have to be resilient against manipulation. To address this, TELEMETRY develops a Distributed Ledger Technology (DLT) based Auditable Data Infrastructure to create immutable, auditable records of events, actions, indicators and reports that are generated by other tools. Its distributed nature adds resilience, and it also allows tools in different locations to contribute to the same records and share data such as test results or alerts among themselves. The Auditable Data Infrastructure in TELEMETRY extends prior work on a platform known as SmartQC [39] which was developed in a smart manufacturing context. 

**Tool Name**: Auditable Data Infrastructure. **Purpose**: Data sharing platform based on DLT, for maintaining an immutable, auditable record of events, alerts and reports generated by the various security and anomaly detection tools. **Features and Benefits**: The platform offers the benefits of DLT, in particular immutability of records shared in a distributed ledger, while abstracting from its complexity by featuring a data sharing API on top that is accessible by the various tools in the TELEMETRY ecosystem. The abstraction layer further allows for flexibility in the choice of underlying DLT based on the features that are required, instead of being limited to one particular platform. **Input**: In relation to testing tools such as the Network Fuzzer or the SBOM Generator, events and reports will be received directly from those tools. In the live operation stage, aggregated reports originating from anomaly detection tools will be received via the SIEA pipeline. **Output**: Through the Client API, tools such as the SSM can query the data sharing platform for reported data. Notifications will be triggered by incoming reports from testing phase tools and will be sent to inform the SSM of new content. **Results**: The Auditable Data Infrastructure can accommodate a selection of underlying DLT flavours such as Hyperledger Fabric [24], IOTA^14^, and BigchainDB [40]. In relation to functionality, one extension to the original platform is the triggering of actions or notifications by certain transactions. When new reports are received from testing tools such as the Network Fuzzer or the SBOM Generator, these may be of relevance to the risk assessment that is being done by the SSM. Hence, the SSM has to be notified of those new reports, meaning that the transaction that commits the report to the ledger should at the same time trigger such notification. Underlying context and meta data structures have been aligned with cybersecurity indicator specifications that are developed in the project.

### Data Aggregation and Filtering

In the TELEMETRY framework, multiple upstream anomaly detection tools will report their findings during operation. It is a task of the ‘Security Information and Event Acquisition’ (SIEA) pipeline to ingest, aggregate, filter and prioritize these security related events and forward compact results to the Auditable Data Infrastructure or downstream tools. This is necessary because some events (e.g. repeated status messages with the same information but only separated by time) flood the message exchange, effectively creating a benign denial of service attack. Therefore, there is a need to filter, aggregate or summarise these events.

**Fig. 5:**
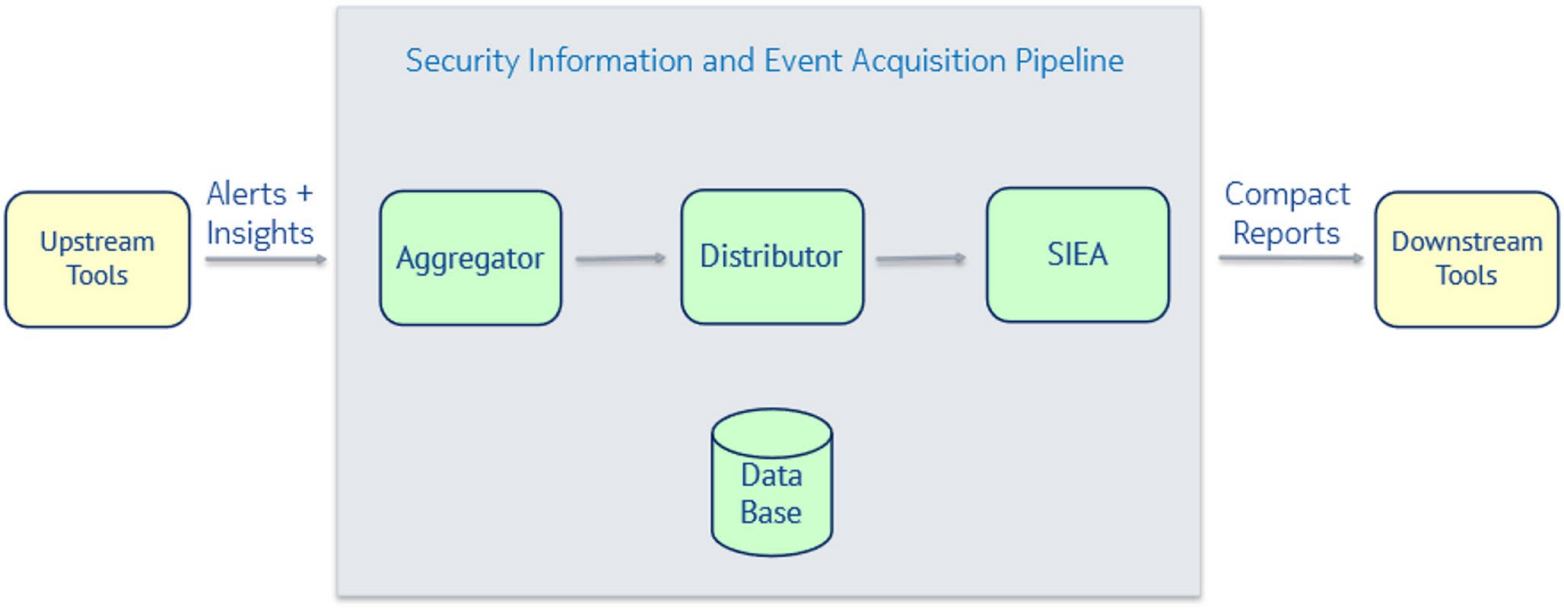
SIEA Pipeline

The SIEA pipeline (Fig. 5) consists of a set of applications: The *Aggregator* subscribes to a message broker and listens to alerts and insights reported by the upstream tools. The Aggregator is aware of the priorities mapped to each security-related event and can therefore control the flow of data to the Distributor. Next to this feature, the Aggregator can enforce a ‘silence period’ if multiple alerts of the same type are reported in high frequency by a ‘noisy’ upstream tool. The *Distributor* is a non-blocking web service capable of buffering events from the Aggregator in case a downstream tool is not ready for ingesting the next chunk of reports. The *SIEA* itself engages in the handshakes of downstream tools guaranteeing the correct processing of the compact reports without overloading the tools themselves. The applications are supported by a database which stores the status of the downstream tools and the corresponding SIEA application for forensics.

**Tool Name**: Security Information and Event Acquisition (SIEA) Pipeline. **Purpose**: Collects, aggregates, filters, and prioritizes security-related events and vulnerabilities. Connects to the downstream components via RESTful southbound interface for further processing. **Features and Benefits**: In order not to flood the downstream tools, the SIEA pipeline groups, aggregates and, in some cases, suppresses recurring events. The Pipeline controls the flow of events to downstream tools via RESTful handshake. The SIEA Pipeline operates in real-time and offers others a controlled connection and managed data flow to its consumers. **Input**: Security related events from monitor tools (described above). **Output**: Prioritized, aggregated and filtered events specific to the upstream tools. **Results**: The Pipeline has been implemented with 4 applications, evaluated with the following lessons learned. The Aggregator must be able to ingest messages from different upstream tools. Based on what they deliver, adaptations are required in the Aggregator itself. Similarly, the SIEA must perform handshakes with different downstream tools. The SIEA is dockerized, which enables it to be setup in different customized instances for each destination tool. The advantages of this approach are a performance boost and an avoidance of blocking situations.

### Workflow Orchestrator

The need for workflows has been established as part of the TELEMETRY work on methodologies, which originated as human-executed methods. The types of tools and the frequency of execution, plus reactive execution of components in response to events, has led to the requirement for automated workflow execution and management.

**Tool Name**: Workflow Orchestrator. **Purpose**: Infrastructure component that configures and executes tools in sequence following workflow templates or user control. **Features and Benefits**: Provides a flexible means of automated tool execution and chaining, enabling different workflows to be specified and triggered either manually or upon specified event conditions. Offers integration with Auditable Data Infrastructure. **Input**: workflow template, tool configurations, trigger conditions, control signals from dashboard. **Output**: Automated execution of tools and storage of results and indicators in Auditable Data Infrastructure. **Results**: An automated workflow engine is in development, and example workflows describing interactions between tools and components are described in the Test Cases Section.

## Test Cases

### Use Case 1: Aircraft Cargo Monitoring

**Fig. 6:**
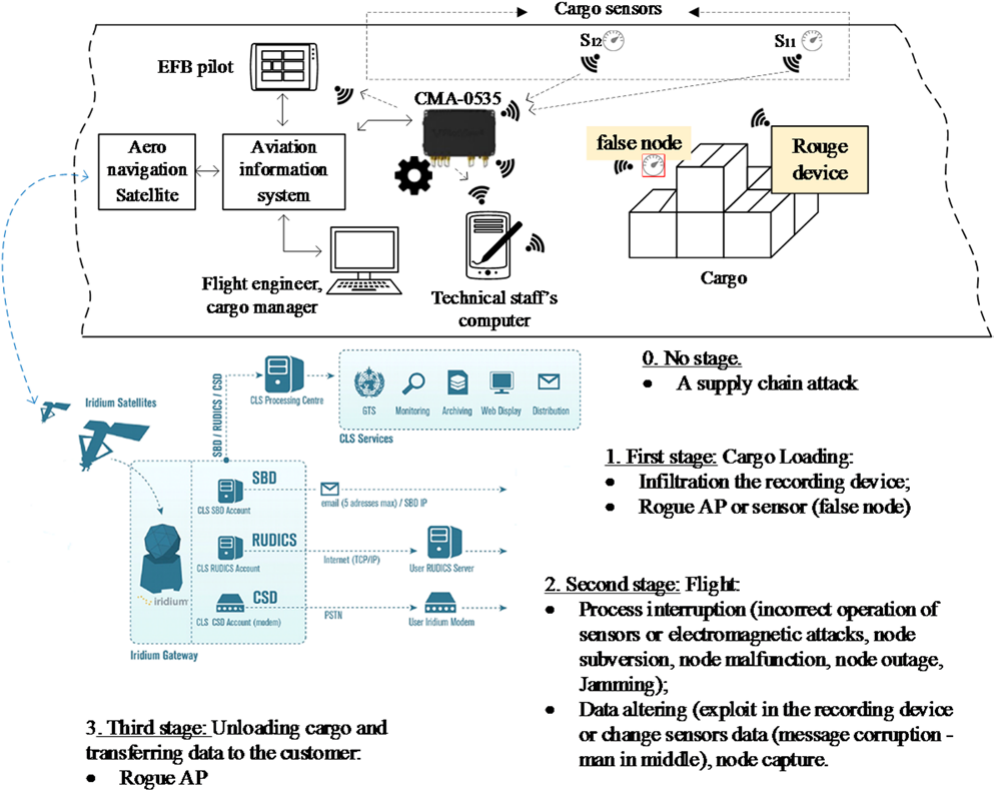
Overview of threats in the aviation use case

Use case 1 focuses on flight cargo monitoring by Antonov Company. The purpose of this is to enable enhanced monitoring of valuable items in the cargo hold and certified transmission of data for live monitoring. The cargo monitoring system monitors the hold visually, whilst recording pressure, humidity, temperature and g-force in three-axes. Sensors are installed with the cargo and the sensors are managed by a special program for monitoring parameters, which is installed on a laptop located in the hold and connected to the local wireless network in the hold. An overview of the scenario, along with the anticipated threats, is shown in Fig. 6.

#### Device Anomaly Detection in Use Case 1

**Fig. 7:**
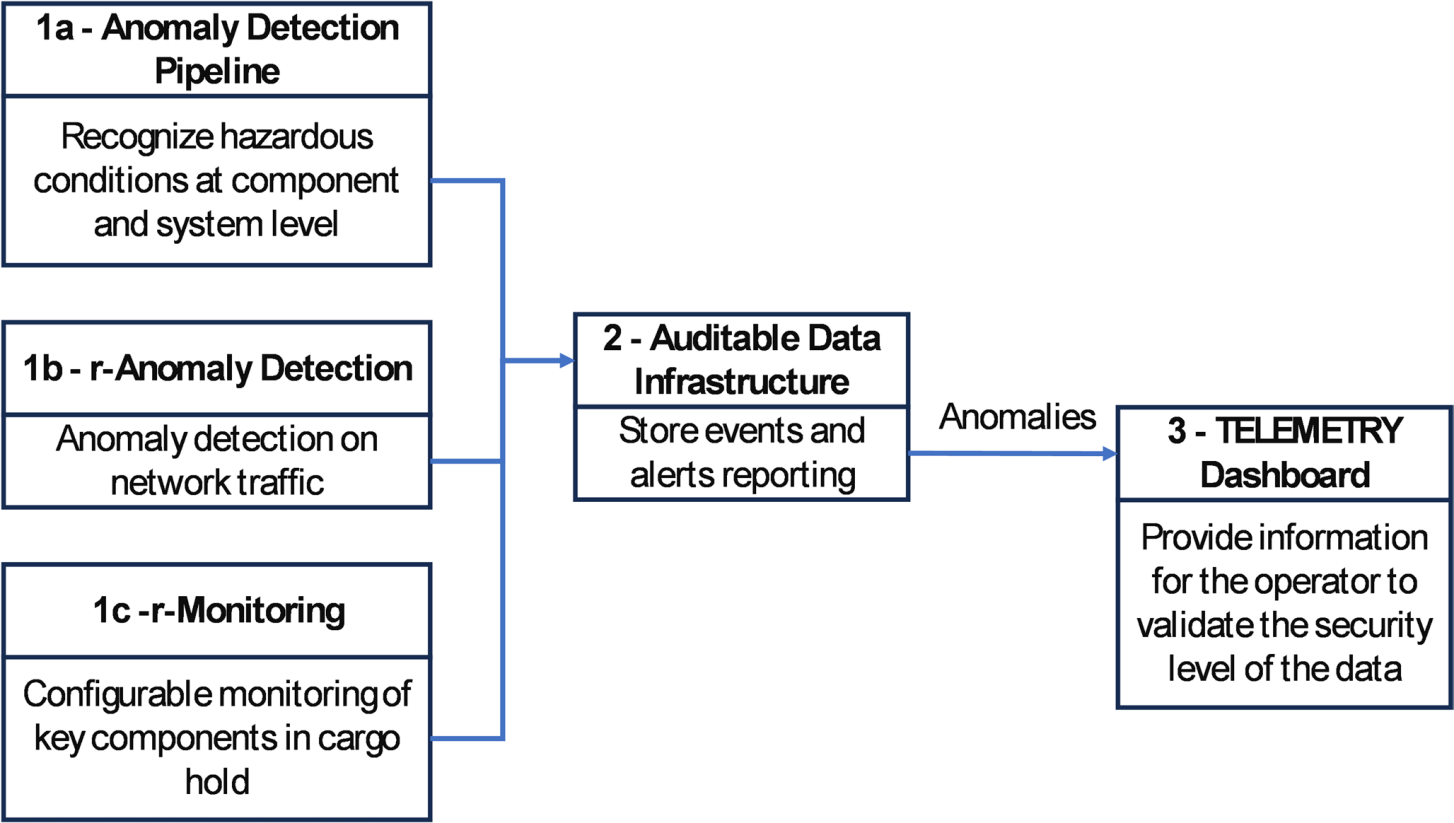
Anomaly detection workflow

The workflow for anomaly detection is shown in Fig. 7. The TELEMETRY tools *r-Anomaly Detection* and *Anomaly Detection Pipeline* help improve the security of an air cargo monitoring system via on-board indicators, information traffic analysis tools and anomaly detection. Within these tools, machine learning tools developed by TELEMETRY partners detect atypical behaviour of onboard information sensors (temperature, pressure, humidity), suspicious network activity and track requests to connect to abnormal network access points, and the results are fed as indicators to the Auditable Data Infrastructure and displayed on the Dashboard. The TELEMETRY tools will be installed on a separate laptop residing in the airplane, and connected to the sensor network also in the airplane. This enables evaluation of the tool with respect to real events.

#### Access Control Risk in Use Case 1

**Fig. 8:**
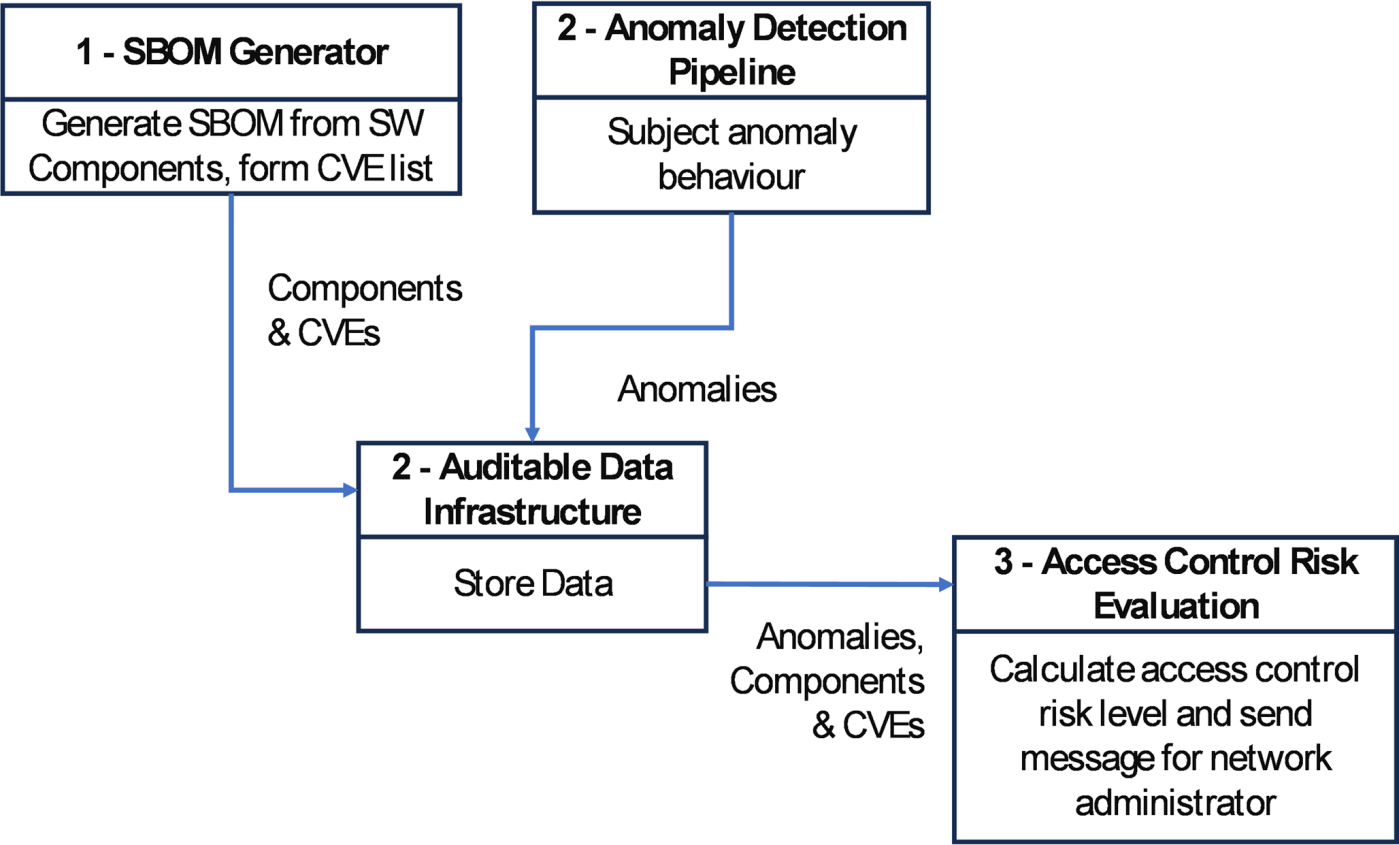
Access control risk workflow

This scenario will identify vulnerabilities and risks in access control policy and enforcement protecting system components that regulate different access levels and sets of rights. The tool for testing access control systems will allow the network administrator to improve the quality of decisions made and reduce response time to incidents. Its workflow is shown in Fig. 8.

### Use Case 2: Smart Manufacturing

Smart factories consist of various interconnected components, such as production machines, network devices, IoT devices, and user devices like laptops and computers. These components, through being ‘smart’ and ‘online,’ are vulnerable to cyber-attacks or could potentially become attackers themselves. Examples of such threats include:

- IoT devices with outdated firmware being hijacked.
- Compromised production machines becoming attackers, possibly via supply chain attacks.
- Unauthorized devices entering the network and launching internal attacks.

**Fig. 9:**
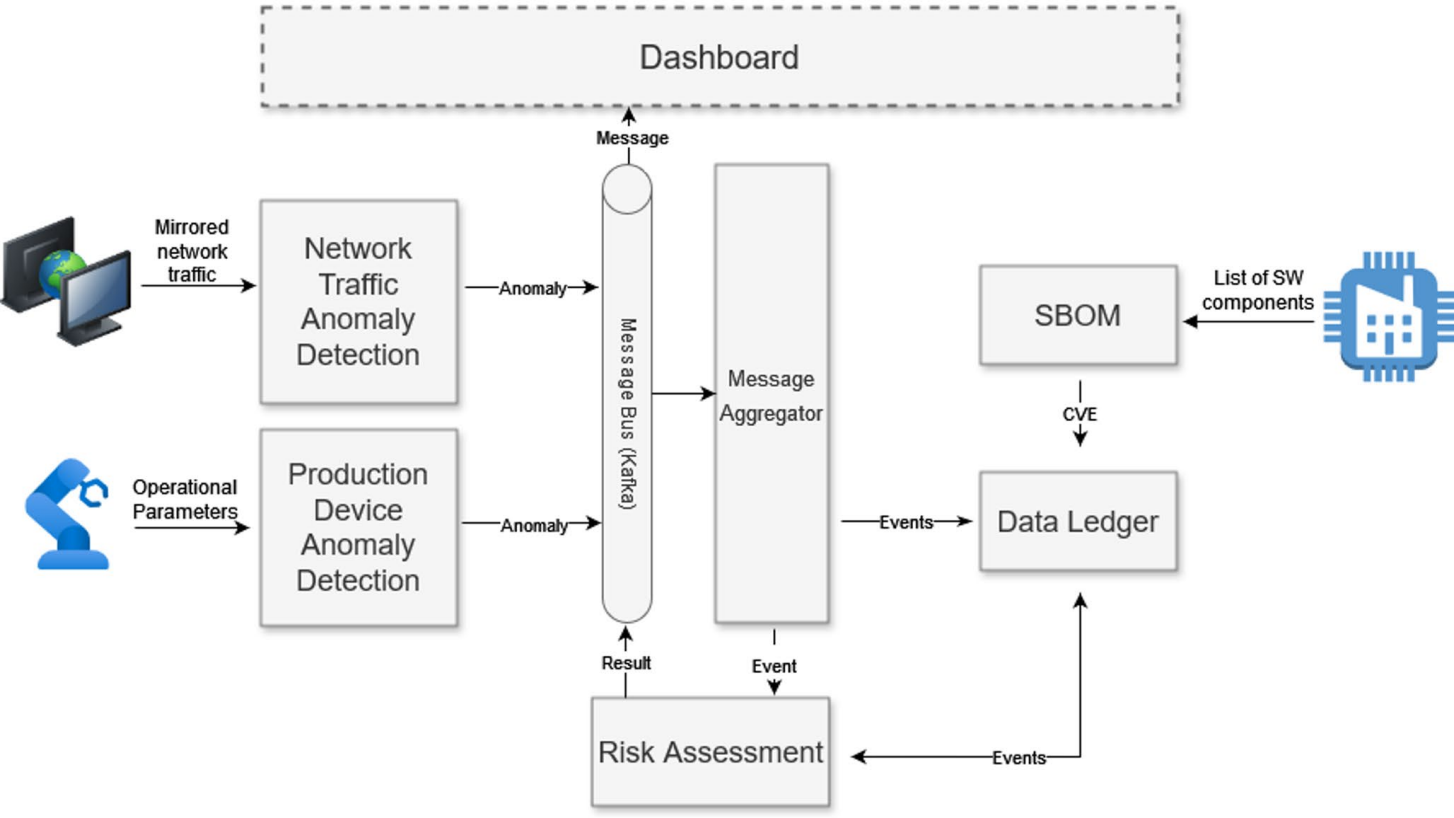
Use case 2 architecture diagram

The approach to addressing these threats is shown in Fig. 9.

**Fig. 10:**
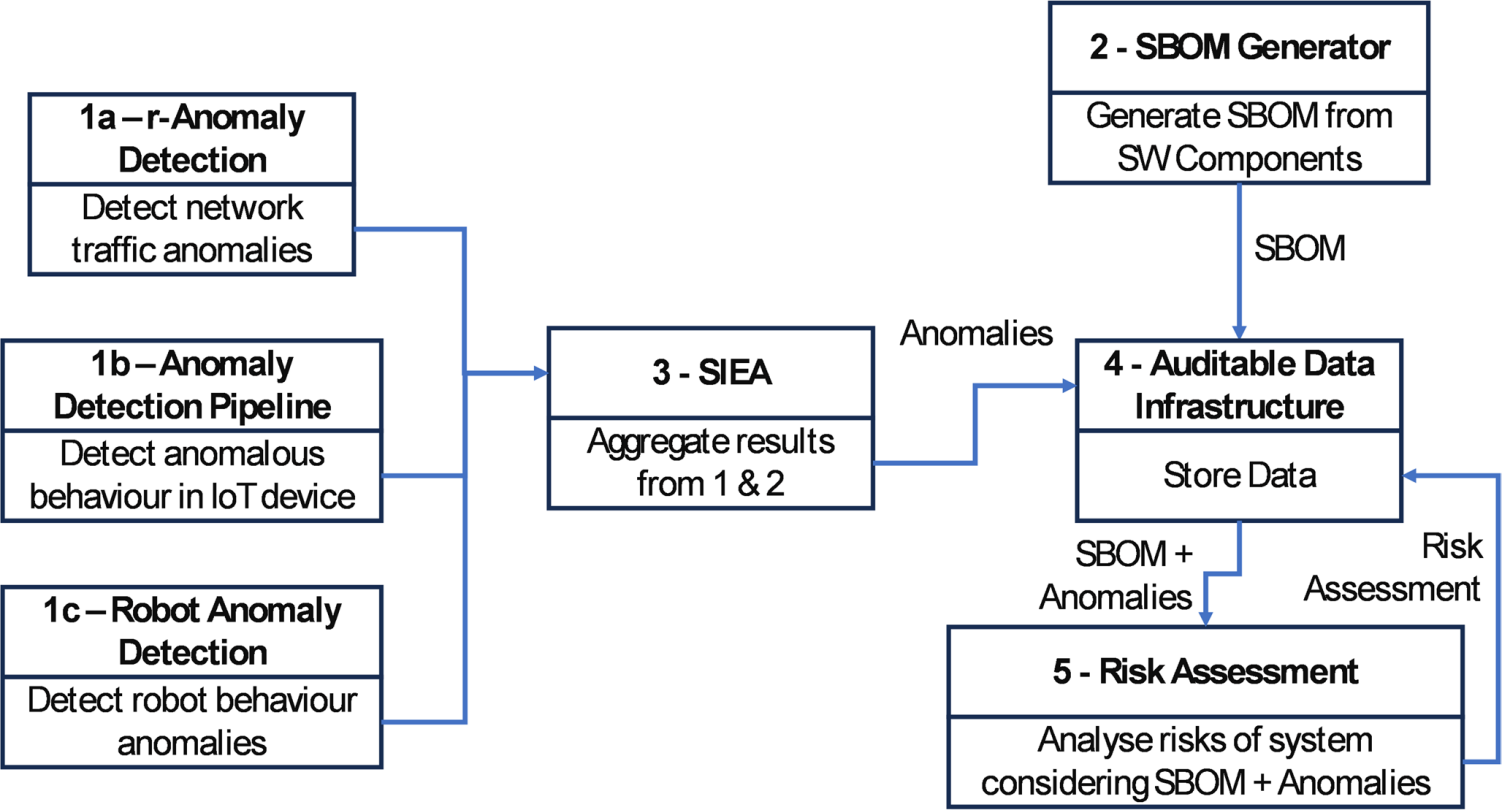
Use case 2 exemplar workflow

Current defence methods like firewalls and traffic scanners are mostly static and only effective against known threats, requiring manual updates. To enhance these defences, TELEMETRY aims to use machine learning to detect unknown cyber-attack variants in real time. For example, machine learning models will analyse robot operating parameters and network traffic to spot anomalies. Additionally, a Software Bill Of Materials (SBOM) will be constructed and compared with vulnerability databases to identify potential exploits. A central risk assessment system will compile these findings, offering a comprehensive risk analysis and recommended countermeasures on a dashboard for administrators. To securely document all events that occur within the smart factory, a distributed ledger technology system is also used, ensuring the integrity and traceability of the recorded data. An exemplar workflow for UC2 is shown in Fig. 10.

### Use Case 3: Telecommunications

Use Case 3 focuses on Residential Gateway (RGW) Devices, also known as home-routers or modem-routers. These devices play a fundamental role, representing the frontier between the customer domain and that of the telecommunications operator. The presence of exploitable vulnerabilities in such devices can indeed have serious consequences, in terms of both access to the operator’s infrastructure and assets (e.g. VoIP platforms, access control systems, remote management solutions, etc.), and of violation of customer data and systems (traffic interception and redirection, access to internal camera streams, access to file repositories, etc.). Furthermore, with the rise of remote working, these devices handle not only personal data but increasingly often also sensitive professional and business information. The number of RGW devices distributed and managed by a single operator can in many cases exceed several millions of units. Finally, despite being low-cost, RGWs are complex devices involving many functions and use multiple third party libraries and thus offer a very large attack surface with multiple physical interfaces (fiber, VDSL, Ethernet, WiFi 5 GHz, WiFi 2.4 GHz, USB, 4G/5G, etc.), active services (DNS, DHCP, SMB, UPnP, DLNA, etc.), all of which represent possible entry points for threats.

**Fig. 11:**
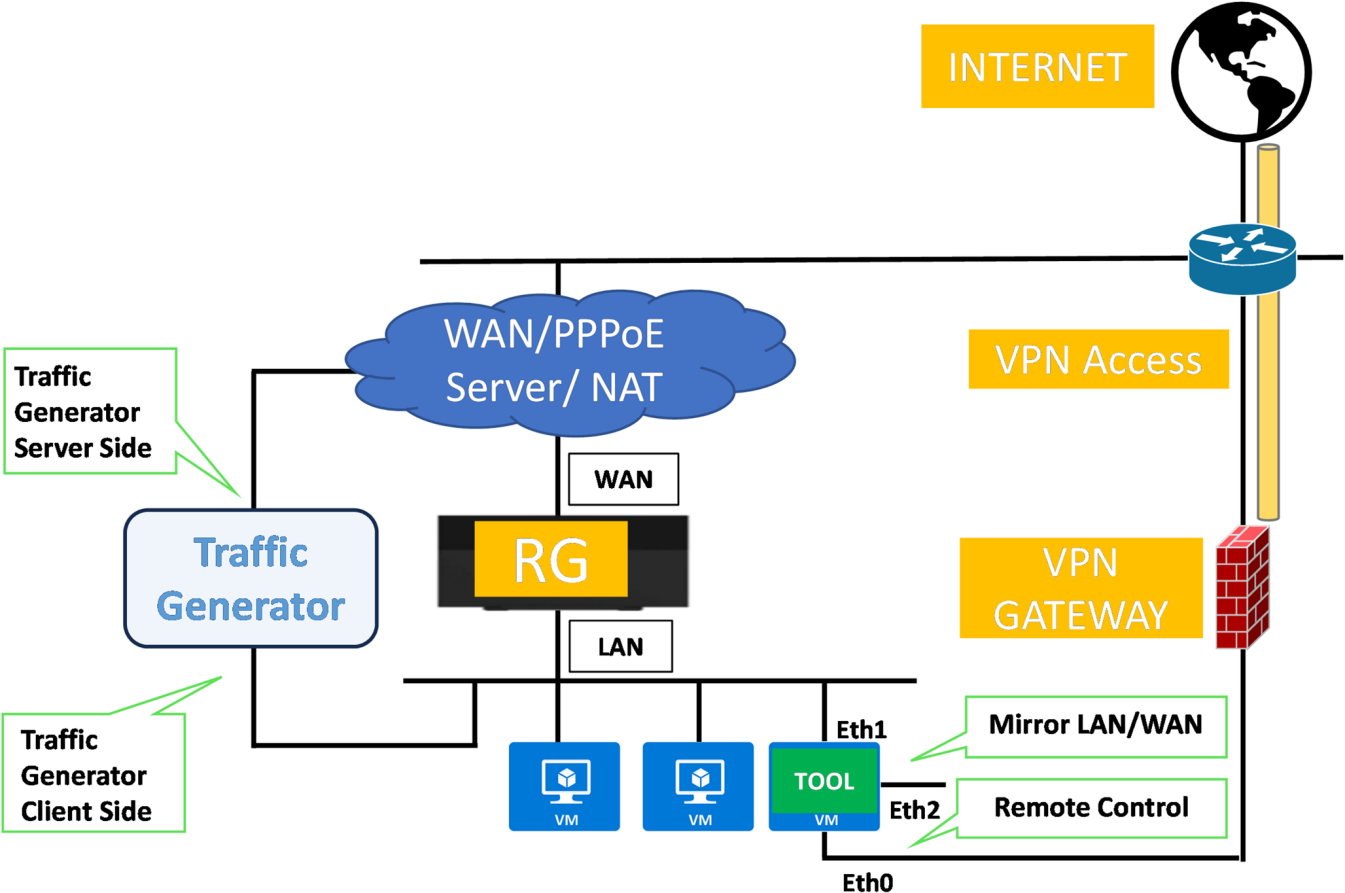
Telco use case testbed

A dedicated test environment simulating real-world conditions has been built (Fig. 11). This includes connecting physical Residential Gateways to both an internal local network (simulating a home environment) and the external Internet. We also added a specialized traffic generator to the internal network, whose role is to reproduce the typical behaviour of users and the applications they use, in order to generate a baseline of legitimate and realistic traffic, needed for training machine learning based anomaly detection tools. The generator is also able to inject attacks or simulate the control phases of compromised hosts. This includes sending packets with spoofed source IP addresses (e.g., for reflection and amplification attacks), executing exploits against vulnerable hosts, establishing cover channels to exfiltrate data (e.g., DNS and ICMP tunnels), creating sessions of anomalous duration, generating traffic during unusual time slots, or, in general, communicating with suspicious IP addresses and domains. TELEMETRY tools are also deployed on the LAN side to collect and monitor traffic data. Additionally, they can capture WAN traffic from the external interface of the RGWs when necessary. In general, the testbed was designed and set up with the primary aims of:

- Reproducing a realistic network environment with typical traffic patterns.
- Hosting the TELEMETRY tools and sensors for development and optimization.
- Providing an environment for effectively evaluating TELEMETRY tools’ ability to test RGWs and generate accurate results.

In the Use Case under examination, numerous challenges must be addressed to mitigate risks, both from the operator’s and the customer’s perspective. Two of these stand out due to their significance and representativeness: prioritizing software and firmware vulnerabilities, typically in the form of CVEs [41], and mitigating/detecting supply chain attacks [42]. Since the former refers to known vulnerabilities and the latter to unknown threats, distinct approaches must be employed by the TELEMETRY tools.

#### CVE Prioritisation

Using vulnerability scanners and other specialized tools, a significant number of vulnerabilities are commonly identified in RGWs. This is generally ascribed to various factors, such as the huge number of software and hardware components within them (applications, services, libraries, drivers, etc.), as well as frequent use of outdated components. For example, the best device among the 117 analysed in Weidenbach and vom Dorp [43] was found to be affected by 348 High severity CVEs relating to the kernel alone, whereas the worst by 579 CVEs. Often, fixing all identified issues is unjustifiable with the time and cost constraints imposed by market demands, time-to-market considerations, or limited fixing capabilities of the device manufacturer. Consequently, it is essential to have tools and methodologies that can be used to concentrate the available resources on fixing vulnerabilities that maximize risk reduction.

To this end, vulnerabilities are typically classified according to the CVSS v3 methodology, promoted by FIRST^15^ and recently updated to version CVSS v4^16^. In the last few years, the CVSS framework has been complemented by other approaches which incorporate dynamic parameters into the severity calculation, like the availability of attack code or active exploitation in the wild. An example of those initiatives is the Exploit Prediction Scoring System (EPSS), also promoted by FIRST^17^ [14]. Although EPSS is a powerful tool for prioritizing vulnerabilities, it presents some limitations and does not consider the actual deployment scenario, such as the context of the specific Use Case under examination.

A further challenge arises from the fact that the RGW firmware provided to the Telco operators is typically customized for the operators and not available as a COTS (Commercial Off-The-Shelf) product. Consequently, each device must be analysed individually which adds to time and effort. Moreover, vulnerability scoring systems such as the Common Vulnerability Scoring System (CVSS) v3 and the EPSS primarily focus on individual vulnerabilities, without considering the combined effects that those vulnerabilities can have on the system. A chain of vulnerabilities in a specific RGW can generate very different consequences depending on the selected starting set, the individual characteristics of the RGW and the system into which it is deployed. In summary, these methodologies, while very interesting and promising for the IT field and for COTS IoT, address neither the possible relationships, dependencies between the actual device’s software modules, nor the access control model (normally in these devices the customers do not have privileged access).

**Fig. 12:**
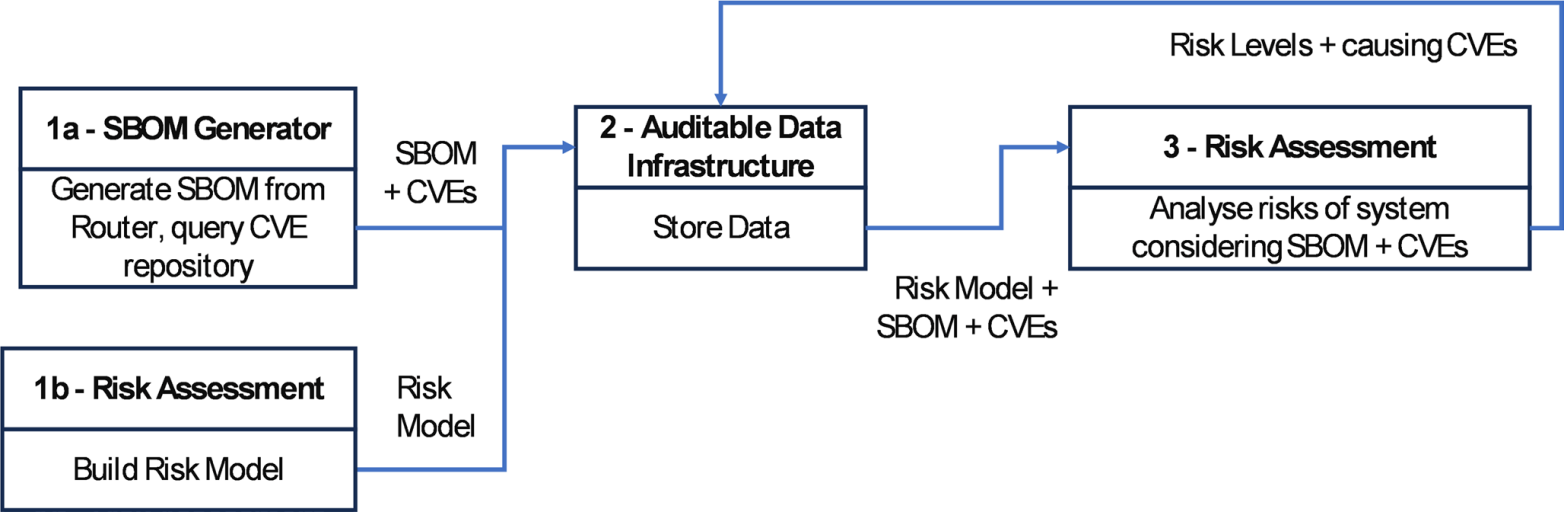
CVE prioritisation workflow

TELEMETRY’s approach to addressing this challenge is described in more detail in Taylor, et al. [15] but summarised here. It involves primarily the SBOM generation and Risk Assessment tools. The SBOM generation tool provides an SBOM for the RGW, the Device Under Test, along with a list of applicable CVEs via query of the NVD database. The Risk Assessment tool has a risk model constructed within it of the router’s hardware and software structure, along with external components that reflect a typical operating scenario (e.g. a user’s home network with other devices, the telco network and the Internet (the screenshot in Fig. 4 above shows an initial version of this risk model). The CVEs detected are mapped to vulnerability parameters within the risk model and risks are generated. The result is a set of attack paths: risks with associated risk levels comprised of impact (severity if the risk occurs) and likelihood, the threats that caused them, and the vulnerabilities (represented by CVEs) that facilitated the threats. Therefore, the tool provides a mapping from risks to CVEs, plus allows easy identification of the CVEs that cause high-level risks. The workflow for this scenario is shown in Fig. 12.

#### Supply Chain Attacks

As defined in Kotolov [42], a supply chain attack is a cyber-attack that seeks damage by targeting and infecting components in the software supply chain, e.g. third-party libraries imported into a codebase. Notable examples include the SolarWinds breach of 2020^18^ and the more recent open-source XZ Utils incident of 2024^19^, where a fake identity was used to infiltrate the GitHub development team as early as 2021. Device firmware contains many commercial and open-source component libraries. An attack on the manufacturer’s infrastructure or a public repository could lead to the inclusion of malicious components on the device which, if undetected, could have serious consequences for the operator and its customers. A malicious component could collect user data, exfiltrate it to external servers, modify device configurations for illicit surveillance (such as altering DNS server IP addresses), compromise internal hosts, and more. Consequently, early intervention during the design/testing phase is crucial, employing tools capable of identifying unforeseen anomalous or malicious behaviours.

The approach to address this uses recursive SBOM generation to determine the libraries and versions used in a DUT and then mapping them to CVEs via lookup. The SBOM generation recurses back up the software supply chain—i.e. an SBOM for a DUT is generated, which returns a manifest of components and associated CVEs. Each of these components must have an SBOM generated for its upstream dependencies (i.e. the libraries the component imports), again with associated CVEs. Each of these dependencies must have an SBOM and CVEs found, and this process must recurse backwards until no upstream dependencies are found.

## Summary of Contributions

The challenges posed by Taylor et al. [1] are addressed by TELEMETRY as follows.

1. There is a need to consider the **full lifecycle of IoT components**—at their design time, their integration into systems, and operation of those systems. As described above in the relationship between the Microsoft SDL and the TELEMETRY framework, the TELEMETRY tools cover the full lifecycle. Typically testing tools cover design, coding and deployment into systems, and monitoring and anomaly detection tools cover operation of devices within systems, but the tools may be used at any stage of the lifecycle as deemed appropriate.
2. **Threats and risks can propagate when components are connected in systems**—vulnerabilities in one component can affect other components in a system. The relationship between the device and the system is addressed via threat propagation in the SSM risk assessment tool, which specialises in transparently describing the effect of devices within systems and impacts vulnerabilities in one component on other components it is connected to in a wider system. The SSM provides analytical tools that enable tracing back from a risk occurring in one component to the threats and vulnerabilities in other components that caused the risk.
3. **IoT devices present limitations to current testing and management** due to geographical distribution, opacity and limited processing power. TELEMETRY tools address this via lightweight monitoring components that may be installed within low power devices. TELEMETRY also provides multiple tools that monitor systems, the behaviour of devices within those systems and raise alarms if the system behaviour is abnormal.
4. **Risk assessment fulfils an important requirement** because it enables assessment of what elements are important to the system’s stakeholders, how these elements may be compromised, and how the compromises may be controlled. TELEMETRY supports two types of risk assessment: ACRAM, a specialised risk assessment tool for access control; and SSM, a cybersecurity risk assessment tool that enables the assessment of devices within systems.
5. Feedback from operational monitoring of IoT devices can inform firmware updates/patches to the devices but there is a significant challenge in **rolling out these patches to multiple low-power devices geographically distributed**. This is directly addressed by the Secure Update Tool, which enables efficient, scalable and lightweight update of distributed and decentralised systems.

This work has resulted in key observations and findings beyond that of Taylor et al. [1], summarised here.

1. **It is a key requirement that the tools can be used in different combinations as decided by the operator.** The Framework’s architecture is therefore intended to be a flexible, extensible framework aimed at providing testing and monitoring tools for IoT and software. The framework comprises testing tools; runtime monitoring, analysis and detection tools; risk modelling tools; security control tools; testing and evaluation environments; and framework infrastructure. Mapping this work to development lifecycles is a useful means of determining applicability and utility of the tools and framework. In this paper we have mapped TELEMETRY to the Microsoft SDL due to its recency and ubiquity as a methodology from a major vendor.
2. **Tools may be used in multiple different combinations**, and ad-hoc use is also encouraged, where **one tool may provide clues and other tools executed to undertake further investigations based on initial results**. Automated execution of tool chains is supported by workflows representing commonly used combinations of tools expressed templates describing sequences of tools, user decision points, data interactions, to address a particular problem.
3. **For audit and compliance purposes**,** there is a need for immutable storage of testing configurations**,** results and other data.** This is addressed via storage and sharing using Distributed Ledger Technology (DLT), which facilitates this immutable storage.
4. **Indicators are observations or measurements representing information of relevance for assessment of cyber security risk**,** such as observable vulnerabilities**,** incidents and threats.** An indicator is an output of one tool or a combination of outputs from multiple tools aggregated together (e.g. the correlation of output values may comprise an indicator). Indicators may enable decisions regarding actions or subsequent tooling.
5. **There is a key interplay between devices and systems (Device Under Test / System Under Test)** and the decision whether an entity under examination is a device or a system depends on perspective and context: **a device can be a system in its own right and might be integrated into a wider system.**
6. **Anomaly detection in multiple forms is a key means of runtime monitoring.** It is implemented using explainable machine learning techniques to pinpoint key indicators. Normal behaviour is identified and recorded, and monitored live observations are compared against it to determine anomaly alarm conditions. A key observation is that deployment in each situation requires customisation/training as the normal behaviour is highly specialised to the deployment conditions, and indeed what is “normal” is likely to change over time, requiring dynamic model adaptation involving moving norms.
7. **Tools’ configuration and usage is highly dependent on the SUT/DUT and the usage context**—there is significant complexity and differentiation between e.g. a router’s software and hardware construction compared with that of a sensor used within an aircraft, so there will be considerable investigation needed related to the specifics of each device/system as an item of further work.

## Further Work

Further work involves finalisation of tool development, further testing each of these approaches and identifying further combinations of tools necessary to the needs and detail of each application area. We will further develop the TELEMETRY workflow concept, elaborating workflows for all the use cases where TELEMETRY tools are demonstrated.

We will also explore how the TELEMETRY tools can be used to satisfy testing requirements of certification processes, e.g., as part of complying with the EU Cyber Resilience Act. We will carry this over into the emerging EU cybersecurity certification schemes, particularly the EU5G scheme that has relevance for telecom operators.

## Conclusions

This paper has presented an overview of the TELEMETRY framework that aims to provide an extensible suite of tools and infrastructure that enables testing and monitoring of ICT ecosystems, with a special focus on IoT devices and their placement within wider systems.

The work presented here addresses the challenges across the whole lifecycles of IoT devices/systems under test (DUT/SUT), at both design time of devices, their integration into wider systems and operation of those systems. System-wide risk assessment tools enable assessment of threat propagation across interconnected devices in systems, informed by indicators provided by device level and system level monitoring components that observe the DUT/SUT. Auditability is considered an important property for compliance with relevant regulations, and we provide an auditable data infrastructure backed up by Distributed Ledger Technology to provide immutable recording of observations, indicators, risks, decisions and actions. IoT devices are by nature low computational power and widely distributed so we provide specialised patching rollout to enable secure, efficient updates to correct any vulnerabilities found.

The framework architecture has been specified, first versions of components have been developed and evaluated in use cases with results fed back for subsequent development. The paper has described the approaches to address the challenges presented in previous work.

## Data Availability

N/A—no data is applicable.

## Footnotes

1
https://dictionary.cambridge.org/dictionary/english/system.

2
https://github.com/jtpereyda/boofuzz.

3 e.g. https://www.comparitech.com/net-admin/pcap-guide/.

4
https://spdx.dev/.

5
https://cyclonedx.org/.

6
https://www.iso.org/standard/65666.html.

7 e.g. https://www.infosecurity-magazine.com/news/us-government-proposes-sbom-rules/.

8 e.g. https://medium.com/@interlynkblog/eu-cra-and-sbom-5100c55752fa.

9
https://nvd.nist.gov/.

10
https://www.qemu.org/.

11
https://github.com/ReFirmLabs/binwalk.

12 CyberRange: Advanced simulation and training solution, https://www.cyber.airbus.com/products/cyberrange/.

13
https://wazuh.com/.

14
https://www.iota.org/learn/intro.

15 Common Vulnerability Scoring System v3.1: Specification Document, CVSS v3.1 Specification Document https://www.first.org/cvss/v3-1/cvss-v31-specification_r1.pdf.

16 Common Vulnerability Scoring System v4.0: Specification Document, CVSS v4.0 Specification Document https://www.first.org/cvss/v4-0/cvss-v40-specification.pdf.

17 Exploit Prediction Scoring System (EPSS), Exploit Prediction Scoring System (EPSS) https://www.first.org/epss/.

18 Jake Williams, What You Need to Know About the SolarWinds Supply-Chain Attack, SANS Institute. https://www.sans.org/blog/what-you-need-to-know-about-the-solarwinds-supply-chain-attack/.

19 Cedric Pernet, XZ Utils Supply Chain Attack: A Threat Actor Spent Two Years to Implement a Linux Backdoor, TechRepublic. https://www.techrepublic.com/article/xz-backdoor-linux/.

## Declarations

### Generative AI

The authors used generative artificial intelligence tools to support the drafting and stylistic refinement of the literature review. The tools were not used to generate original scientific claims, analyses, or interpretations. All cited sources were selected, verified, and interpreted by the authors, who assume full responsibility for the content of the manuscript.

### Research involving human and /or animals

N/A—no research involving humans or animals was undertaken in this paper.
